# Parallels between drought and flooding: An integrated framework for plant eco‐physiological responses to water stress

**DOI:** 10.1002/pei3.10117

**Published:** 2023-06-30

**Authors:** Siluo Chen, Kirsten H. W. J. ten Tusscher, Rashmi Sasidharan, Stefan C. Dekker, Hugo J. de Boer

**Affiliations:** ^1^ Computational Developmental Biology, Department of Biology Utrecht University Utrecht The Netherlands; ^2^ Centre for Complex System Studies Utrecht University Utrecht The Netherlands; ^3^ Plant Stress Resilience, Institute of Environmental Biology Utrecht University Utrecht The Netherlands; ^4^ Environmental Sciences, Copernicus Institute of Sustainable Development Utrecht University Utrecht The Netherlands

**Keywords:** drought, ethylene, flooding, modeling, oxygen deficit, soil‐plant‐atmosphere continuum, water stress

## Abstract

Drought and flooding occur at opposite ends of the soil moisture spectrum yet their resulting stress responses in plants share many similarities. Drought limits root water uptake to which plants respond with stomatal closure and reduced leaf gas exchange. Flooding limits root metabolism due to soil oxygen deficiency, which also limits root water uptake and leaf gas exchange. As drought and flooding can occur consecutively in the same system and resulting plant stress responses share similar mechanisms, a single theoretical framework that integrates plant responses over a continuum of soil water conditions from drought to flooding is attractive. Based on a review of recent literature, we integrated the main plant eco‐physiological mechanisms in a single theoretical framework with a focus on plant water transport, plant oxygen dynamics, and leaf gas exchange. We used theory from the soil–plant–atmosphere continuum modeling as “backbone” for our framework, and subsequently incorporated interactions between processes that regulate plant water and oxygen status, abscisic acid and ethylene levels, and the resulting acclimation strategies in response to drought, waterlogging, and complete submergence. Our theoretical framework provides a basis for the development of mathematical models to describe plant responses to the soil moisture continuum from drought to flooding.

## Graphical abstract

Our study aims to develop a conceptual framework to describe plant responses across a range of soil moisture conditions that span from drought to flooding. We use theory from the soil–plant atmosphere continuum modeling as “backbone” for our framework, coupling plant oxygen status, and the mechanistic processes of abscisic acid and ethylene signaling to describe plant responses to drought, waterlogging, and complete submergence. Our theoretical framework provides a basis for the development of mathematical models to describe plant responses to the soil moisture continuum from drought to flooding.

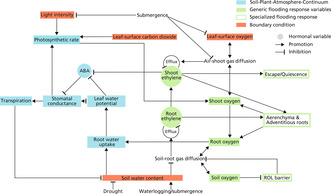

## 1 INTRODUCTION

Rooted terrestrial plants are highly sensitive to the water status of the soil in which they grow, with too little soil moisture causing drought stress whereas excess soil moisture and (partial) submergence causes flooding stress. These stress responses to opposing water conditions can be understood from the fact that the functioning of these plants requires sufficient liquid water uptake from the soil as well as rapid exchange of CO_2_ and oxygen with the environment to enable photosynthesis and support aerobic respiratory processes in the roots, stems, and leaves (Kozlowski, 1984). Crucially, excess soil moisture and (partial) submergence limit the gas diffusion between the plant and its environment.

Insufficient soil moisture results in reduced root water uptake and is typically followed by stomatal closure to limit water loss through transpiration (Sperry, 2000). Stomatal closure subsequently also limits CO_2_ diffusion across the stomata and thereby photosynthesis under conditions when light and other factors are not limiting assimilation of CO_2_ (Farquhar et al., 1982). Depending on the extent of stomatal closure and the drought severity, this condition may lead to carbon starvation or result in vascular dysfunction due to embolism (Adams et al., 2017). Under conditions of excess soil moisture, oxygen diffusion through the soil is limited and root oxygen status declines over time to trigger a transition from aerobic to anaerobic root metabolism at a specific threshold (Kosmacz & Weits, 2014). This decline in root metabolism limits root water transport while prolonged root anoxia may cause root decay or even plant death (Colmer et al., 2014). Despite the apparent opposing character of these two water stresses, both conditions result in a reduction of plant water transport, photosynthesis, and transpiration at the leaf level and, therefore, have similar consequences for fluxes of water and carbon between the vegetation and atmosphere.

Environmental conditions associated with drought and flooding can occur consecutively in the same system in any order. To understand and predict responses of individual plants and ecosystems to periods of drought and flooding and propose mitigating measures enhancing ecosystem robustness in future climates, the development of models capable of operating across this range of conditions is essential. Moreover, continuous development of the land surface component of climate models leads to the inclusion of more detailed plant eco‐physiological processes which are relevant for the surface exchange of water, carbon, and energy (e.g., Fisher & Koven, 2020; Harrison et al., 2021). However, a model with sufficient mechanistic biological details needed to simulate the interrelated changes in plant water transport, gas exchange, and photosynthesis to this range of hydrological conditions is, to our knowledge, currently lacking.

The aim of our paper is to develop an integrated mechanistic framework for a model that can describe plant responses across a continuum of moisture conditions that range from drought through waterlogging to submergence, building on the biophysics of plant water transport and gas exchange and its dependence on environmental conditions. Hereto, we review the major known eco‐physiological responses of plants to drought, waterlogging, and submergence, with a specific focus on mechanisms that govern plant water transport and gas exchange. Key variables to be integrated into such a framework are stomatal conductance, shoot and root oxygen content, as well as shoot and root ethylene levels, given that ethylene mediates a series of acclimation mechanisms in flood‐tolerant plants, such as aerenchyma formation and adventitious root development, as well as “escape” and “quiescence” strategies under submergence (Voesenek & Sasidharan, 2013).

In this paper, we first describe the soil–plant–atmosphere continuum, which describes the biophysical processes of water movement in plants, as an eco‐physiological basis for the development of our mechanistic framework and define the main conditions that occur from drought to flooding. We then briefly review the main plant eco‐physiological responses during drought conditions. Subsequently, we derive the mechanisms needed to describe these key plant responses across the moisture continuum with an emphasis on responses that occur during waterlogging and submergence. We specifically focus on feedback between acclimation responses in our mechanistic framework and highlight the resulting similarities between drought and flooding responses. We end with a discussion focused on potential research questions that can be addressed with the proposed mechanistic framework.

## 2 THE SOIL–PLANT–ATMOSPHERE CONTINUUM FROM DROUGHT TO FLOODING

Water transport is an essential aspect of plant life and a major factor through which plants affect our climate. There is thus a rich scientific tradition aimed at understanding and modeling plant water transport. The most widely acknowledged plant hydraulics theory thus far is the “cohesion‐tension theory” (Tyree, 2003). This theory indicates that transpiration makes use of the capillary force created by the hydrogen bonds between water molecules (cohesion) and between water molecules and cell walls (adhesion) to create “tension,” a negative (sub‐atmospheric) pressure throughout the water column. Thus, the water molecules are pulled out in the xylem, and due to the cohesion, the other water molecules are continuously pulled along the tension gradient from the roots to the leaves. This results in the generation of a water potential gradient along the xylem, with a lower (more negative) potential at the canopy and a higher (less negative) potential at the roots (Steudle, 2001). A typical model that describes plant water transport is the soil–plant–atmosphere continuum model (Elfving et al., 1972). The model makes use of a so‐called “Ohm's analogy,” essentially treating water flow as current and the soil–plant–atmosphere system as a series of resistances. The water potential of each plant compartment (i.e., root, stem, and canopy) together with the resistance between these compartments determine the amount of water flow (Bonan, 2019). The soil–plant–atmosphere continuum model is usually coupled with a photosynthesis model via stomatal behavior to simulate plant hydraulics and leaf‐level gas exchange processes during normal growth conditions and drought (Bonan et al., 2014). Therefore, we propose the soil–plant–atmosphere continuum‐photosynthesis model to serve as the “backbone” of our model framework, which is then extended to couple plant biochemical processes in response to flooding as well.

**FIGURE 1:**
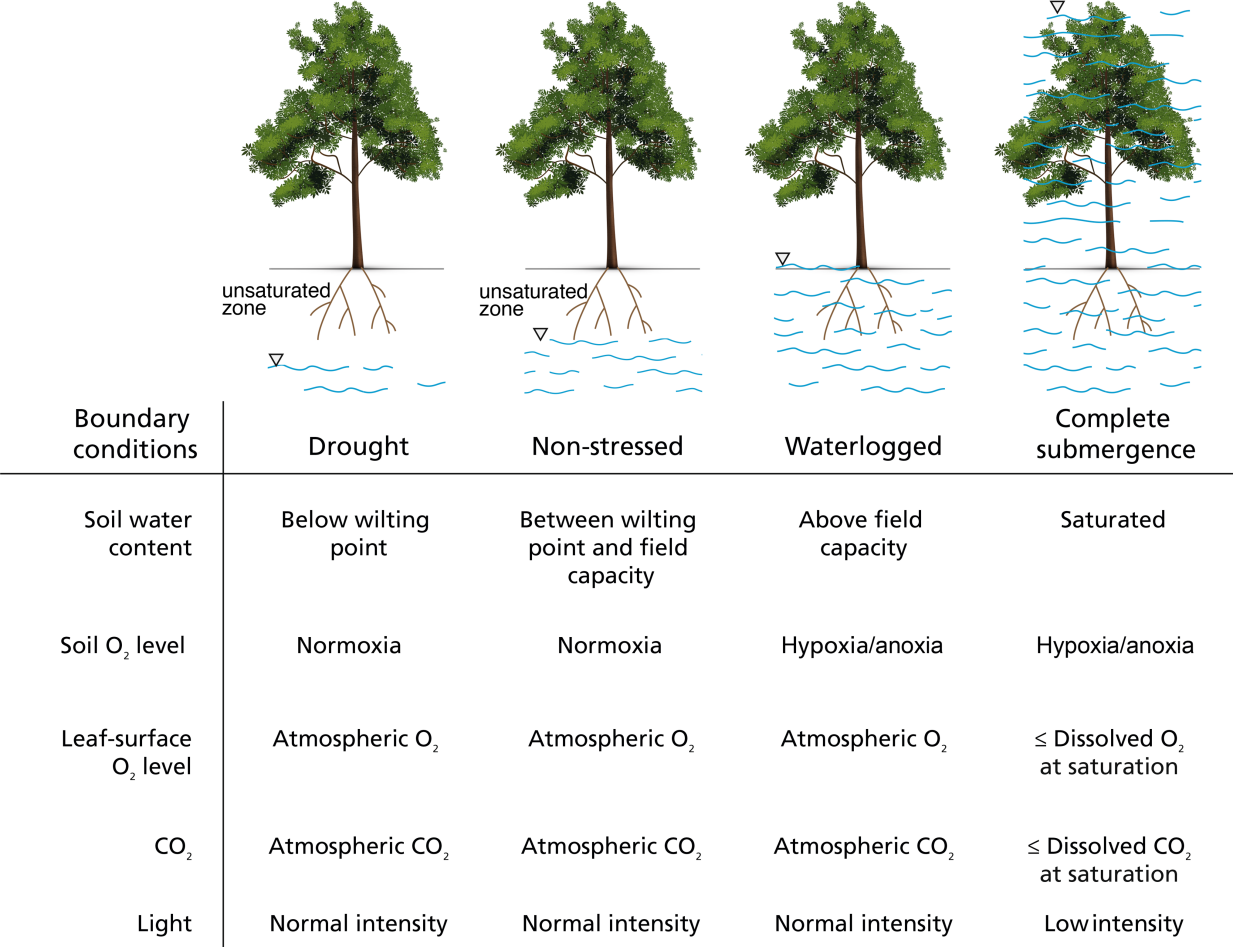
The boundary conditions in terms of environmental boundary conditions (i.e., soil water content, soil oxygen level, leaf‐surface oxygen level, leaf‐surface CO_2_ level, and leaf‐surface light intensity) under drought, non‐stressed, soil waterlogged, and completely submerged conditions.

In our review and theoretical framework development, we discuss four distinct scenarios, which, in the order of increasing wetness are drought, non‐stressed condition, waterlogging (sometimes referred to as soil flooding (M. B. Jackson & Armstrong, 1999)), and complete submergence (Figure 1). Under non‐stressed conditions, the soil water content is by definition between the wilting point and the field capacity. The soil oxygen state is generally below atmospheric level but still has sufficient supply for root functioning (O'Connell et al., 2018), and is thus considered as normoxia, while the shoot ambient oxygen and CO_2_ are at atmospheric level, and light intensity is uninhibited during daytime. During drought, soil water content is below the wilting point which means that available soil water for plants is lacking, while the aeration status of root and shoot and the available light intensity are similar to those during non‐stressed conditions (O'Connell et al., 2018). Waterlogging and complete submergence are two distinct states of flooding. During waterlogging, soil water content exceeds field capacity, and soil pores are nearly or fully filled with water while the shoot remains exposed to air and light (Sasidharan et al., 2017). Gas diffusion coefficients in water are typically 1/10000 of that in air (Armstrong, 1980), and dissolved oxygen in saturation is at a very low concentration of 8.3 mg L^−1^ under 25°C and 1 atm pressure. In this case, the soil is severely hypoxic and the root functioning is disturbed (Parent et al., 2008), whereas the above‐ground shoot remains aerated. During submergence, the soil is waterlogged and hypoxic like waterlogging, and the aboveground shoot is also underwater. The available light intensity depends on the turbidity of the floodwater (Vervuren et al., 2003). Photosynthesis under floodwater with low turbidity tends to be sustained, while that under highly turbid floodwater is often largely inhibited (Mommer et al., 2005; Oladosu et al., 2020). Owing to the theoretical complexity beyond the scope of the intended application of our theoretical framework, we only discuss the submergence scenario with high turbidity, and thus do not consider specific mechanisms involved in underwater photosynthesis, despite its importance for understanding some plant responses to submergence (Pedersen et al., 2013). Meanwhile, plants in our proposed framework are assumed to be mature, therefore, processes related to life history are also not considered.

## 3 INTEGRATING ECO‐PHYSIOLOGICAL RESPONSES FROM DROUGHT TO FLOODING

### 3.1 Generic responses to drought

**FIGURE 2:**
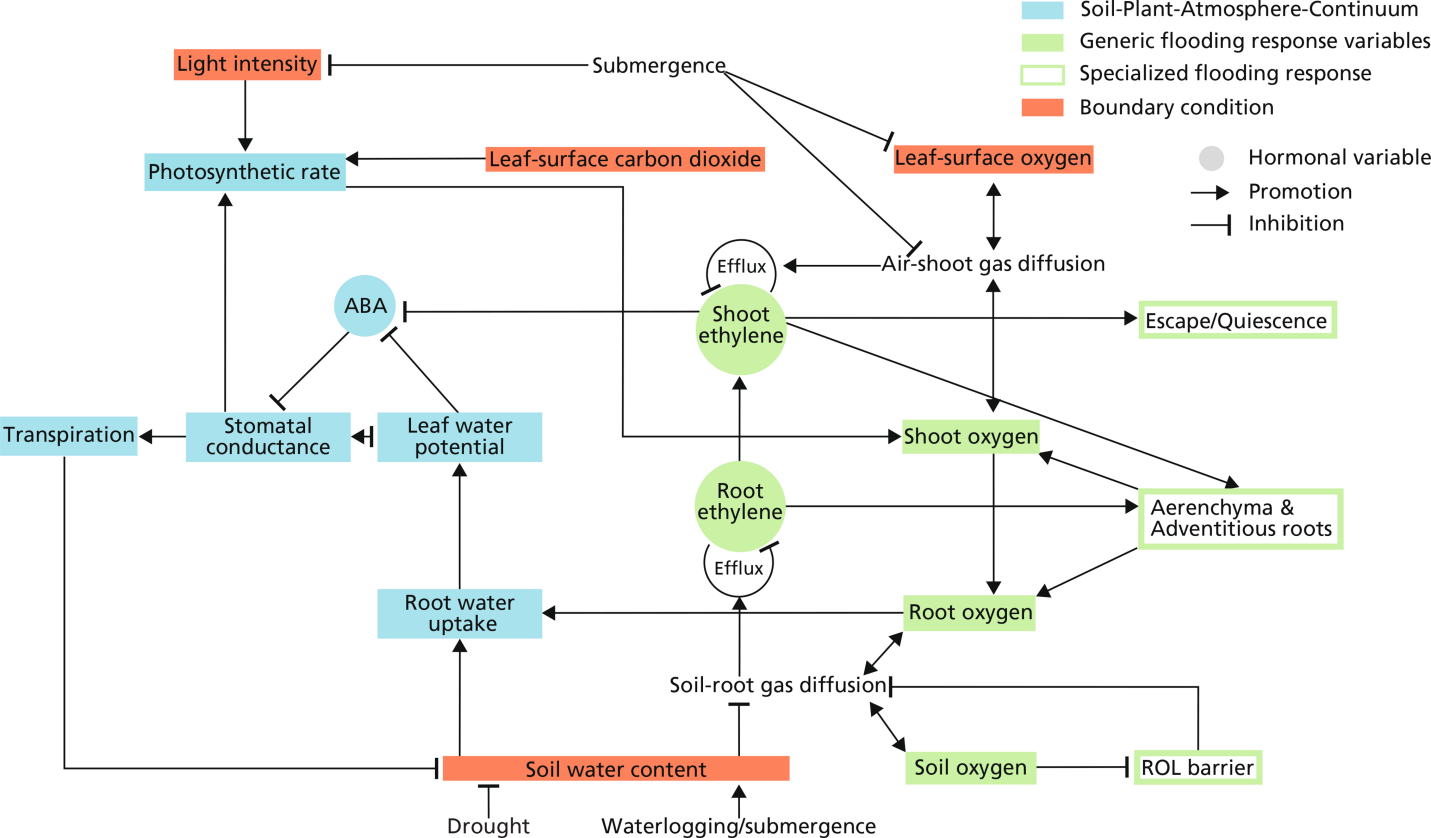
Conceptual framework to describe plant responses across a range of soil moisture conditions that span from drought to flooding. The framework integrates responses involving changes in plant water status, plant oxygen status, and hormonal signaling ABA and ethylene. During drought conditions, plant water status is regulated through passive (hydraulic) and active (hormonal) stomatal control pathways. The passive hydraulic pathway of stomatal control is represented by that soil water deficit causes a reduction in root water uptake, leading to more negative leaf water potential and reduced stomatal conductance. The active hormonal pathway of stomatal control is represented by the reduction of leaf water potential induces ABA biosynthesis and signaling that reduces stomatal conductance. During waterlogging conditions, excessive soil water prevents the gas diffusion between soil and root, limiting the root oxygen level, which then relies on the shoot oxygen supply. Root oxygen deficit then causes the gating of aquaporins, thereby limiting root water uptake through a reduced root water conductance. This mechanism links the flooding response to the drought response. Oxygen deficit also induces the local biosynthesis of ethylene, and due to that reduced soil‐root gas diffusion limits the ethylene efflux, root ethylene abruptly accumulates after flooding. When there is complete submergence with high‐turbid floodwater, photosynthesis is largely impeded due to reduced light tensity and leaf‐surface CO_2_. The floodwater also entraps the oxygen and ethylene inside the shoot due to the impeded air‐shoot gas diffusion, causing an abrupt increase in shoot ethylene. Ethylene mediates a series of specialized flooding response strategies in flood‐tolerant plant species, including aerenchyma and adventitious root development, and the employment of “escape” and “quiescence” strategies under submergence. Examples of these flood‐tolerant plant species and their corresponding acclimation strategies are briefly summarized in Table 1. Ethylene also inhibits ABA biosynthesis and signaling, which serves as another link between the drought and flooding response. The reduced air‐shoot gas diffusion from floodwater entrapment also impedes transpiration, thereby shutting down the soil–plant–atmosphere continuum system.

The plant response to drought has been well captured by existing soil–plant–atmosphere continuum models (Elfving et al., 1972; Manzoni et al., 2014; Zhang et al., 2021), in which the key processes are outlined in Figure 2 (blue). In response to the soil water deficit, plants regulate their stomatal aperture to restrict water loss and abate the decline of their water status (Buckley, 2005). This process is generally implemented via two pathways—passive hydraulic control and active hormonal control, which are additive in effect (McAdam & Brodribb, 2014). The passive hydraulic control involves the reduction of hydraulic conductance from soil to leaf (Müllers et al., 2022), which mostly results from the air gaps between soil and root (North & Nobel, 1997), the gating of aquaporins (Domec et al., 2021), root suberization (Kim et al., 2022), or xylem embolisms (Martínez‐Vilalta & Garcia‐Forner, 2017; Tyree, 2003). The limited whole‐plant water transport and the leaf‐level transpiration demand will lower leaf water potential (Ehrler et al., 1978), with resulting guard cell turgor loss and stomatal closure (Brown et al., 1976). Active hormonal control occurs when leaf water potential drops below a certain level, causing leaf turgor loss and triggering the biosynthesis of abscisic acid (ABA) (Cardoso et al., 2020; McAdam & Brodribb, 2014; Sussmilch et al., 2017). ABA is able to trigger a series of processes that eventually leads to membrane polarization and potassium ion efflux, causing further guard cell turgor loss resulting in enhanced stomatal closure (Hauser et al., 2017). The resulting reduced stomatal conductance causes a lower transpiration rate and a lower photosynthetic rate. This is based on Fick's law of diffusion that the amount of gas exchange (i.e., water vapor diffusing out and CO_2_ diffusing in) is determined by stomatal conductance and the partial pressure deficit inside and outside the leaves.

Plant species differ in their tendency to decrease stomatal conductance in response to drought stress. On the one end of the spectrum are plant species using a so‐called isohydric strategy (e.g., maize and pea) (Bates & Hall, 1981; Tardieu, 1993) in which plants produce more ABA and as a consequence substantially reduce their stomatal conductance during soil water dry‐down (Coupel‐Ledru et al., 2017), thus maintaining a relatively high (less negative) and stable leaf water potential but sacrificing photosynthesis rate (Bonan, 2019). On the other end of the spectrum are plant species employing an anisohydric strategy (e.g., soybean and wheat) (Allen et al., 1994; Henson et al., 1989), in which less ABA is produced in vascular tissues (Coupel‐Ledru et al., 2017), and plants maintain relatively high photosynthesis rate by keeping their stomatal conductance relatively stable, resulting in leaf water potential becoming very negative and thereby rendering the plant prone to desiccation in case of prolonged drought (Bonan, 2019).

### 3.2 Generic responses to waterlogging and complete submergence

The fundamental plant physiological stress that results from soil waterlogging is root oxygen deficit, which forces a switch from aerobic to anaerobic root metabolism (Kozlowski, 1984). According to the so‐called Pasteur effect, to generate the same amount of ATP, about 15 times as much glucose is required in anaerobic respiration as in aerobic respiration, and plants are therefore prone to mortality from energy exhaustion. Meanwhile, root oxygen deficit causes dysfunction of root water uptake due to gating of aquaporins (Törnroth‐Horsefield et al., 2006). This is the key process through which the soil–plant–atmosphere continuum compartment and the flooding response compartment (green in Figure 2) are coupled in our theoretical framework. As the shoot remains well‐oxygenated due to exposure to the atmosphere, the loss in root water uptake, similar to the case under drought stress, can then lead to the imbalance between transpiration demand and limited internal plant water transport (Aroca et al., 2012). Therefore, water potential in root, stem, and canopy becomes more negative, and xylem water conductance is reduced (Ashraf, 2012; Nicolás et al., 2005). To reduce the resulting risk to desiccation of the above‐ground tissues due to excess transpiration, plants downregulate their stomatal aperture which ultimately results in a lower photosynthesis level (Ahmed et al., 2002; Jackson & Drew, 1984; Liu et al., 2014). Although oxygen can diffuse from shoot to root, the overall flux is limited to the high resistance of low‐porosity tissues (Armstrong & Armstrong, 2014). Additionally, the waterlogged root suffers from radial oxygen loss to the anoxic soil (Armstrong, 1971). These effects further trap roots in hypoxic and even anoxic conditions under prolonged waterlogging.

Under complete submergence, as the turbid flood waters can severely limit light availability and this in combination with limited gas diffusion largely impedes underwater photosynthesis. In such cases, the shoot oxygen mainly comes from the direct diffusion from the floodwater. Logically, plant water transport in the soil–plant–atmosphere continuum system is halted during submergence as there is no longer an atmospheric demand driving the evaporation flux.

### 3.3 Specialized flooding responses—The “escape” and “quiescence” strategy

**FIGURE 3:**
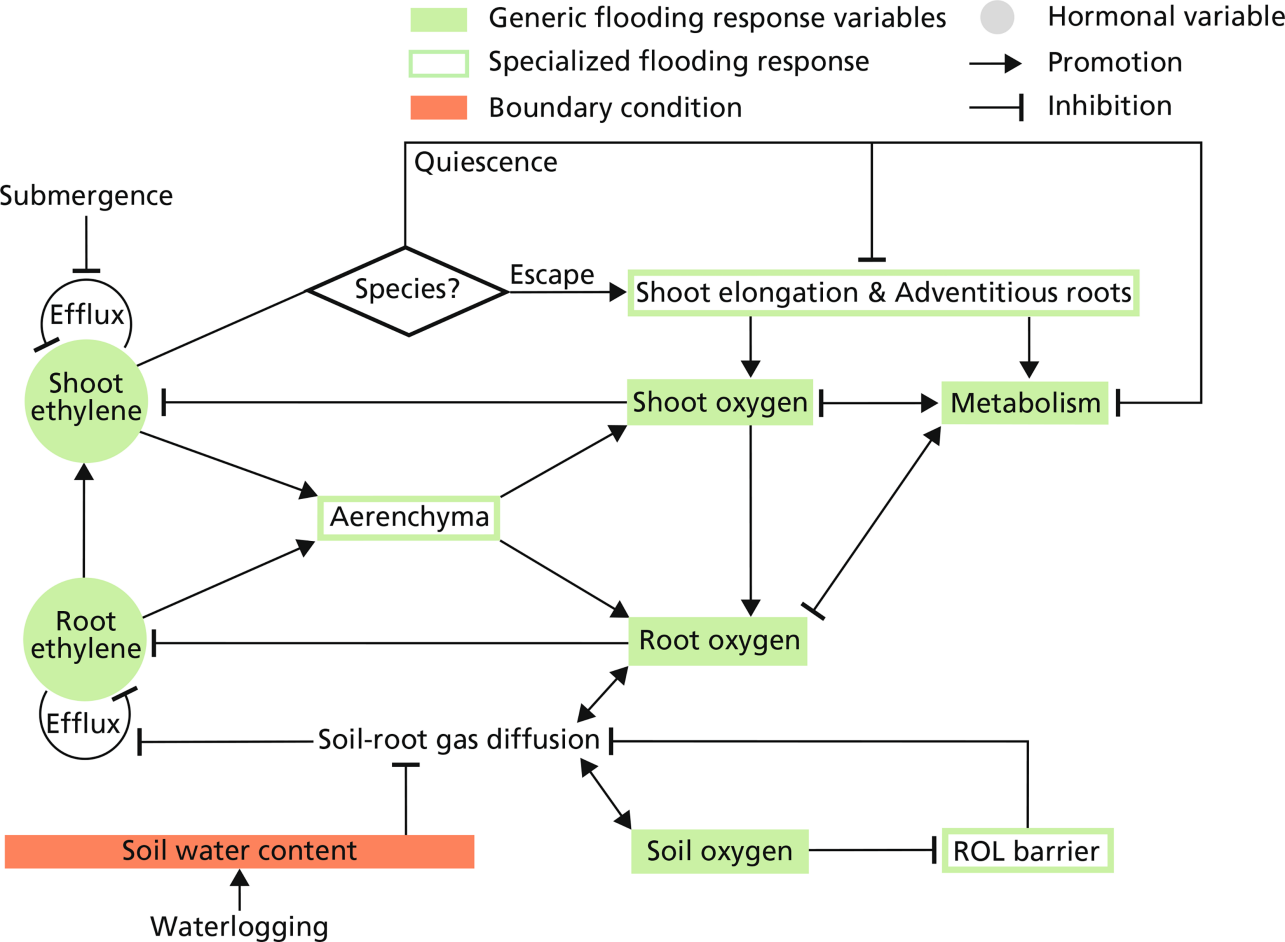
Schematic of the main processes involved in the specialized flooding response, characterized by aerenchyma formation, ROL barrier formation, adventitious root development, shoot elongation, and the “quiescence” strategy. Shoot and root ethylene increases due to the entrapment by floodwater and the ethylene biosynthesis induced by low oxygen levels. Under complete submergence, depending on the species and ecotype, plants employ either “escape” strategy, characterized by developing aerenchyma‐rich adventitious roots and shoot elongation, or “quiescence” strategy, characterized by a downregulation of metabolism and energy‐consuming activities.

Flood‐tolerant plants have developed specialized acclimation mechanisms to survive waterlogging and complete submergence. In Figure 2, we briefly illustrated the induction and effect of these acclimation mechanisms, of which the processes are more detailedly depicted in Figure 3. Ethylene serves as a principle phytohormone that mediates these mechanisms (Shiono et al., 2008; Voesenek & Sasidharan, 2013). Ethylene is a gas, and during flooding its outward diffusion is largely inhibited by the surrounding floodwater (Stünzi & Kende, 1989), causing an abrupt increase of endogenous ethylene in submerged plant tissues (Sasidharan et al., 2018). Endogenous ethylene accumulation induces the expression and stabilization of ethylene response factor (ERF) VII transcription factors that trigger low‐oxygen acclimation mechanisms (Bailey‐Serres et al., 2012; Van Dongen & Licausi, 2015). There are two major submergence survival strategies, typically referred to as “escape” and “quiescence.” The escape strategy is observed in deep‐water rice and involves accelerated internodal elongation to facilitate shoot emergence above water. This shoot elongation promotes plant endogenous oxygenation and maintenance of photosynthesis through aerenchyma formation and adventitious root development (Bailey‐Serres & Voesenek, 2008). In contrast, the quiescence strategy is conservative and energy‐saving (Pradhan & Mohanty, 2013) and involves the repression of energy and carbon consumption, including growth, thereby promoting the maintenance of carbohydrates and energy reserves (Voesenek & Bailey‐Serres, 2015). We have included brief descriptions of these specialized responses in our conceptual model displayed in Figure 2. A summary of the common flood‐tolerant species that employ these strategies is listed in Table 1. Further details on the processes involved in these specialized flooding responses will be discussed in the following section.

**TABLE 1:** Examples of plant species employing specialized flooding responses.

Flooding‐response strategy	Condition	Example species	Reference
Aerenchyma & adventitious root	Waterlogging	Maize Deep‐water rice Soybean	(Yamauchi et al., 2016) (Colmer, 2003) (Ploschuk et al., 2022)
ROL barrier	Waterlogging	Teosinte Deep‐water rice	(Abiko et al., 2012) (Colmer, 2003)
“Quiescence”	Complete submergence	Low‐land rice Lotus tenuis* *Rumex acetosa*	(Fukao et al., 2006; Xu et al., 2006) (Manzur et al., 2009) (Van Veen et al., 2013)
“Escape” with shoot elongation and aerenchyma formation	Complete submergence	Deep‐water rice *Rumex palustris*	(Kende et al., 1998) (Voesenek et al., 2003)

*Note*: *Lotus tenuis* can switch between the “escape” and “quiescence” strategy according to the depth of submergence (Manzur et al., 2009).

Ethylene accumulation in waterlogged roots can mediate aerenchyma and adventitious root formation. Ethylene accumulation can activate the plasma membrane‐located respiratory burst oxidase homolog (RBOH) protein that converts molecular oxygen to apoplastic reactive oxygen species (ROS) (Steffens, 2014). The apoplastic ROS further leads to programmed cell death in the parenchyma and epidermis, inducing aerenchyma formation and adventitious root emergence, respectively (Steffens, 2014). The aerenchyma can largely reduce the resistance to plant internal gas diffusion and thus promote plant internal oxygen diffusion from shoot to root. Adventitious roots serve as an aerated root system alternative to the waterlogged primary root system; it is aerenchyma‐rich, and can therefore transport oxygen at low resistance (Voesenek & Bailey‐Serres, 2015). The development of aerenchyma‐rich adventitious roots takes approximately 3–7 days after the onset of flooding (Brailsford et al., 1993; Guan et al., 2019). Root oxygen deficiency upon flooding promotes the accumulation of the ethylene precursor 1‐aminocyclopropane‐1‐carboxylic acid (ACC) (Rodrigues et al., 2014) via upregulation of ACC synthases. In addition, oxygen depletion limits the oxidation of ACC to ethylene (Vanderstraeten & Van Der Straeten, 2017). Ethylene produced in the shoots of waterlogging plants is linked to ACC transported from hypoxic roots and is associated with the induction of various shoot‐level acclimation processes (Bailey‐Serres & Voesenek, 2010; Voesenek & Bailey‐Serres, 2013).

Besides ethylene‐induced aerenchyma formation and adventitious root development, some plant species can also induce a radial oxygen loss (ROL) barrier, a suberin‐rich structural layer at the exodermis to prevent radial oxygen loss from the root to the waterlogged soil (Table 1). Although ROL barrier formation is found in plant species that are able to form aerenchyma, the controlling pathway of ROL barrier formation is ethylene‐independent (Shiono et al., 2011). Instead, it is triggered by the reductive phytotoxins produced by anaerobic microorganisms (Pedersen et al., 2021), such as Fe^2+^ (Mongon et al., 2014), sulfide (Armstrong & Armstrong, 2005), and some organic acids (Armstrong & Armstrong, 2001). In order to constrain the complexity of our framework, in Figure 3 the induction of ROL barrier is drawn directly downstream of soil oxygen deficit.

In plant species displaying the submergence escape strategy, petiole/internode elongation is triggered by the accumulation of shoot ethylene which induces gibberellin signaling (gibberellin not shown in Figure 3) (Fukao et al., 2006). With the aerenchyma formed in the elongated shoot and adventitious roots (Bailey‐Serres & Voesenek, 2008), oxygen can diffuse via the emergent shoots to the root, thus maintaining energy production through aerobic metabolism and root functioning. In deep‐water rice, ethylene mediates this via induction of the *SNORKEL* (*SK*) locus encoding two ethylene inducible group VII ERFs, *SK*1 and *SK*2, (Hattori et al., 2009; Mittal et al., 2022; Nishiuchi et al., 2012). Adventitious root development and shoot elongation require input of energy and carbohydrates (Voesenek & Bailey‐Serres, 2015). Therefore, for the low‐land rice ecotype that is often exposed to flash flooding and prolonged submergence, energy and carbohydrate reserves of plants can hardly afford to “escape” the floodwater, and thus the “quiescence” strategy is preferred (Ismail, 2018). In this ecotype the *SUBMERGENCE* 1 (*SUB*1) locus encodes a group VII ERF, *SUB*1A, induced by accumulated shoot endogenous ethylene, and which represses energy and carbohydrate consumption, including the “escape” processes via gibberellin signaling (gibberellin not shown in Figure 3) (Xu et al., 2006). *SUB*1A also enhances ethanolic fermentation, further promoting the conservation of energy and carbohydrates (Kuroha & Ashikari, 2020). The induction of “quiescence” strategy and its relationship with plant metabolism and “escape” strategy is also displayed in Figure 3.

### 3.4 Eco‐physiological feedback during flooding

**FIGURE 4:**
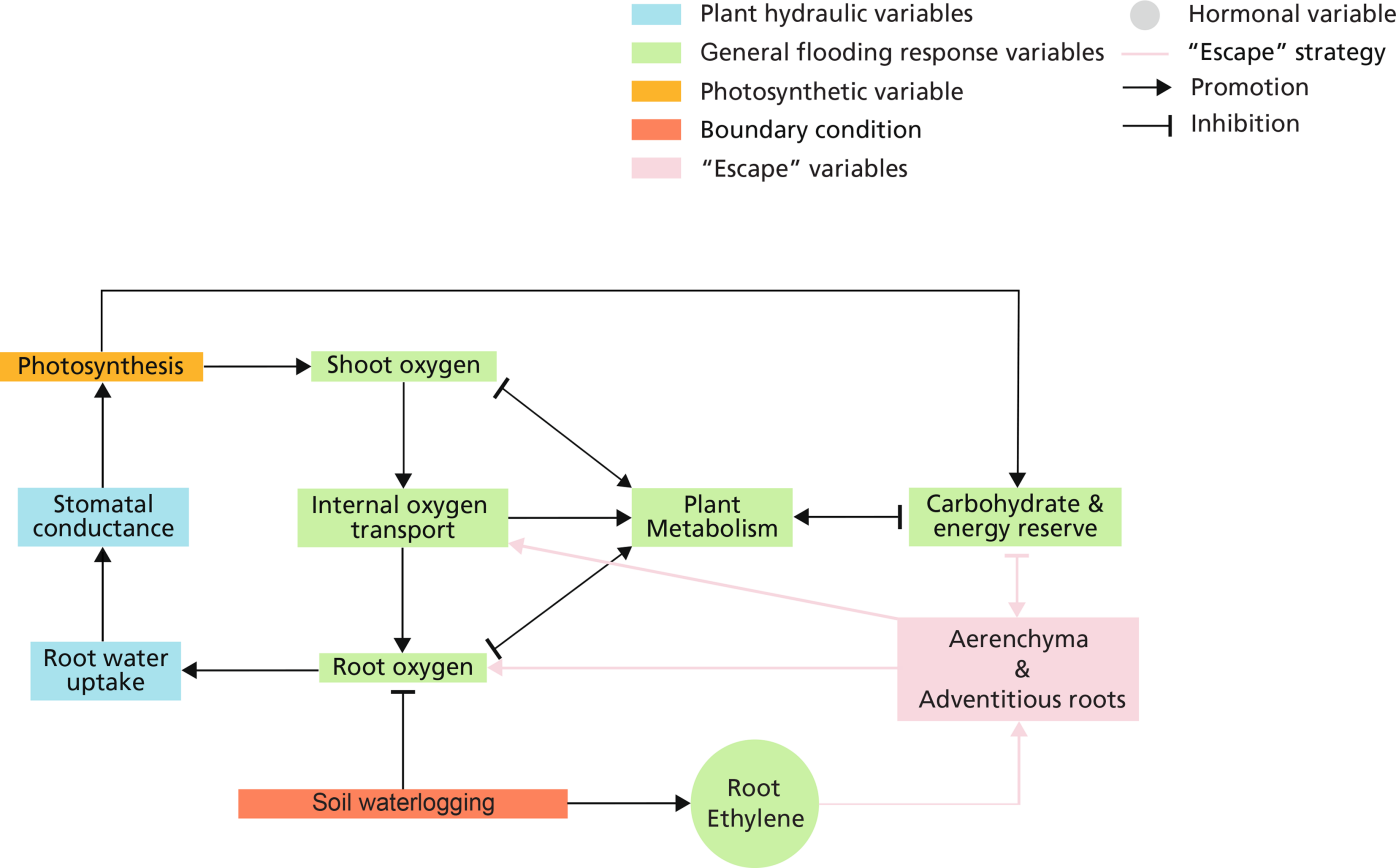
The feedback loop of plant response to waterlogging conditions, coupled with plant hydraulics and photosynthesis. Feedback related to non‐tolerant plant responses are shown in black arrows, the “escape” strategy (i.e., aerenchyma‐rich adventitious root development) shown in the pink box and pink arrows imposes an overall negative feedback loop on root oxygen that serves as the self‐rescuing mechanism.

Finally, we consider how feedback between the various processes involved in flooding responses result in the acclimation of flood‐tolerant species. For non‐tolerant plant species under waterlogging conditions (Figure 4), the root suffers from oxygen deficit whereas the shoot is fully aerated. Still, photosynthesis is reduced as root hypoxia causes reduced root water uptake and lowered stomatal conductance (Bradford & Hsiao, 1982; Toral‐Juárez et al., 2021). The resulting reduced photosynthesis limits carbohydrate production, which is required for plant metabolism. Even though shoots remain normoxic, roots might still suffer severe hypoxia with very limited shoot‐root oxygen diffusion in the absence of aerenchyma (Pedersen & Colmer, 2014). Energy production is then facilitated by the switch to anaerobic metabolism, although it is much less efficient (Parent et al., 2008). For waterlogging‐tolerant plant species, the acclimation processes through ethylene signaling can serve as a negative feedback loop that ameliorates root oxygen conditions (pink arrows in Figure 4). In these species, root ethylene accumulation resulting from the entrapment by the waterlogged soil triggers aerenchyma formation and adventitious root development, promoting shoot‐root oxygen diffusion and providing an oxygenated alternative root system, thereby serving as self‐rescuing mechanisms.

**FIGURE 5:**
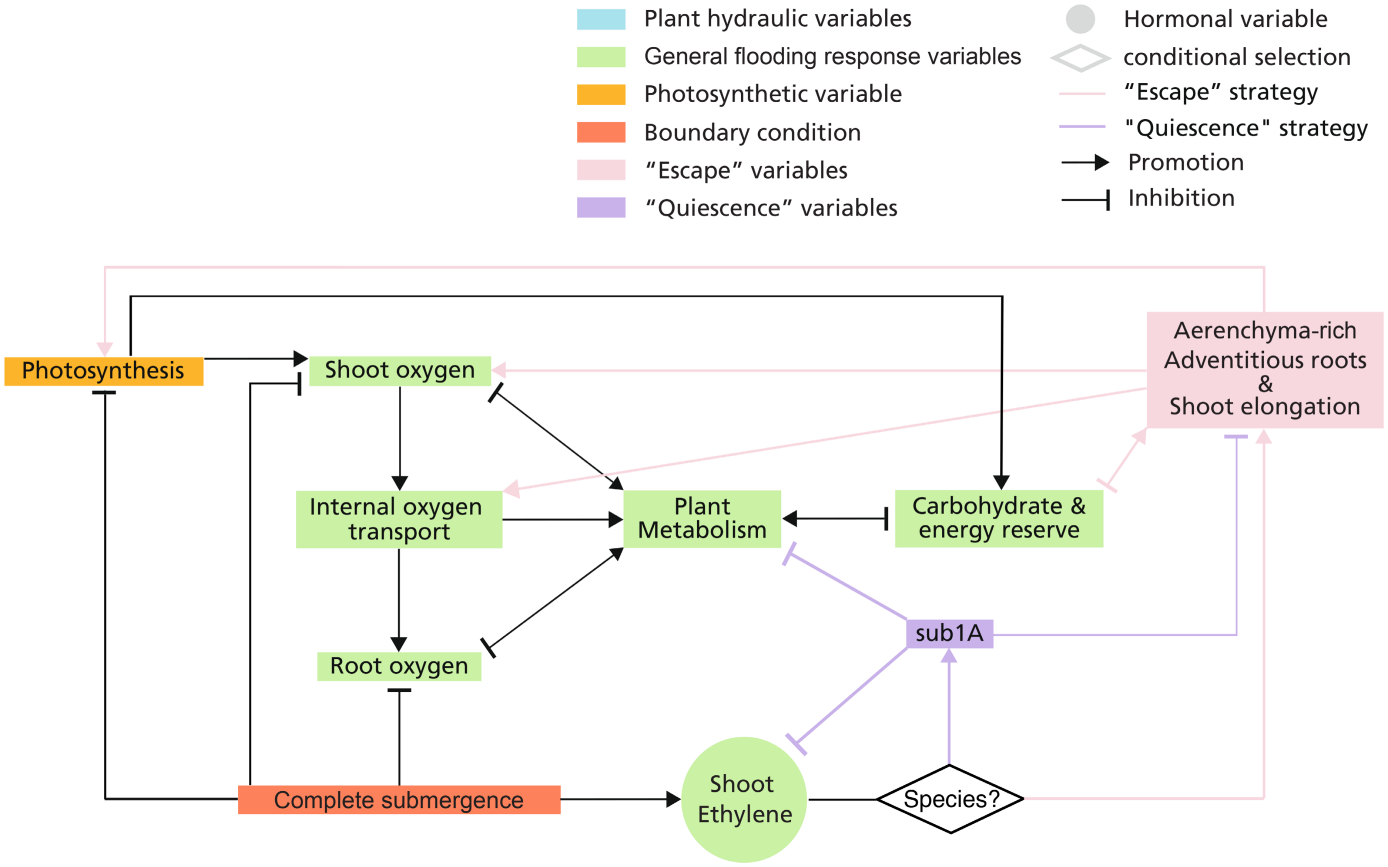
The feedback loop of plant response to complete submergence. Here we consider floodwater with high turbidity so that the light intensity for underwater photosynthesis is limited. The general plant responses shown (black arrows) illustrate plant oxygen deficiency during complete submergence and even carbon starvation. The “escape” strategy (pink box and pink arrows) imposes an overall negative feedback loop on shoot oxygen but an overall positive feedback loop on carbohydrate and energy reserve, while “quiescence” strategy (purple box and purple arrows) imposes an overall negative feedback loop on carbohydrate and energy reserve. Depending on the species and ecotype, plants can either self‐rescue through “escape” strategy or self‐preserve through “quiescence” strategy.

Plant responses to complete submergence are shown in Figure 5. The responses of non‐tolerant plant species are displayed with black arrows, including the inhibition of oxygen diffusion from the ambient environment to both root and shoot, the largely impeded photosynthesis due to high turbidity and limited CO_2_, and a transition from aerobic to anaerobic metabolism (Mommer et al., 2005). Shoot‐root oxygen diffusion is low due to the high resistance to gas diffusion resulting from the low tissue porosity. Consequently, root oxygen deficit is further exacerbated by limited shoot‐root oxygen diffusion, which can reach anoxia within 24 h after shoot hypoxia (Sasidharan & Voesenek, 2015).

Submergence‐tolerant plants can, depending on the species and/or ecotype, either self‐rescue through an “escape” strategy or self‐preserve through a “quiescence” strategy. Both strategies are mediated by shoot endogenous ethylene, which quickly accumulates due to floodwater entrapment. In plants that typically employ the “escape” strategy, a self‐rescuing negative feedback loop can be completed through shoot elongation promoting maintained photosynthesis, shoot‐derived adventitious roots promoting shoot oxygen levels, and aerenchyma promoting shoot‐root oxygen diffusion (pink arrows in Figure 5). In parallel, as shoot elongation and adventitious root development consume carbohydrate and energy reserves, also an exacerbating positive feedback loop occurs. In plants that typically employ a “quiescence” strategy, self‐preservation arises shoot ethylene triggering *SUB*1A expression that represses shoot ethylene biosynthesis, gibberellin signaling, and energy and carbohydrate consumption (Das et al., 2005) (purple arrows in Figure 5).

## 4 DISCUSSION, CONCLUDING REMARKS, AND OUTLOOK

Our proposed model framework considers the soil–plant–atmosphere continuum as a “backbone,” to which hormone‐mediated flooding‐response processes can be coupled to extend the theoretical framework to flooding scenarios, including waterlogging and complete submergence. Efforts of integrating flooding responses to plant hydraulic and photosynthetic models have been made previously. Compared to these efforts, for example, the model developed by Feddes (1982) that phenomenologically simulated stomatal activity in response to soil water content from drought to waterlogging and extended by more process‐based knowledge on oxygen stress (e.g., Bartholomeus et al. (2008)), our proposed model framework attempts to step further by introducing biological mechanistic processes operating under both drought and flooding stress. These processes include oxygen dynamics in the root and shoot and consequences for metabolism in these organs. Also, we include hormonal signaling processes, in which the central flooding hormone ethylene accumulates in affected tissues within a couple of hours after waterlogging or submergence, prior to drop in oxygen levels. The hormonal signaling processes resulting in either “escape” or “quiescence” that are centered on the regulation of gibberellic acid and ABA production and signaling impose a negative feedback loop upon the main skeleton or abate the positive feedback loop of the main skeleton, resulting in a self‐rescuing mechanism or self‐preservation mechanism, respectively.

Following the proposed theoretical framework, mathematical models can be developed to simulate plant responses to the soil moisture continuum from drought to flooding. Different acclimation strategies, such as the discussed an/isohydry in drought response and “escape” and “quiescence” strategies in flooding response can be modeled by varying the sensitivity coefficients that govern key responses. An/isohydry can be simulated by varying the sensitivity of ABA production and stomatal aperture to leaf water potential as previously performed by Tardieu et al. (2015). “Escape” and “quiescence” strategies can be simulated by varying the sensitivity of the extent of aerenchyma formation, adventitious root development, shoot elongation, and plant oxygen consumption in response to ethylene accumulation. For instance, rice is known to have higher aerenchyma content than maize under waterlogging (Pedersen et al., 2021). Therefore, in the model, the sensitivity coefficient that controls aerenchyma induction in response to ethylene of rice can be assigned with a higher value compared to that of maize.

As the plants in the proposed framework were assumed to be mature, except for adventitious root development and shoot elongation that serve as specialized flood‐response morphological developments, the growth of root and shoot is currently not considered. Furthermore, we included a simplified set of hormonal signaling processes. The plant stress response signaling network involved is highly complex, involving a diverse set of hormones, secondary messengers, and genes, between which there is a complex network of crosstalk (Sasidharan et al., 2018). Classically the major phytohormones are subdivided into two categories, the stress response hormones ABA, ethylene, salicylic acid (SA), and jasmonate (JA), and the growth hormones gibberellin, cytokinin, and auxin (Verma et al., 2016). Phenotypically plastic stress responses often arise through the crosstalk between these two categories of hormones. For instance, auxins play a key role in root development, and their transport and signaling are affected by ABA under drought (Rock & Sun, 2005), while under flooding stress, ethylene directly and/or indirectly affects gibberellin signaling, triggering “escape” or “quiescence” strategies, respectively (Bashar et al., 2019). Here we propose to model the dynamics of the stress response hormones and secondary messengers explicitly, incorporating their effects on auxin, cytokinin, and gibberellin only implicitly. Specifically, we propose to incorporate ABA, ethylene, and ROS since the stress hormones JA and SA mainly function during biotic stresses (Verma et al., 2016).

Given the aforementioned simplifications, we suggest that the proposed framework can be used to model short‐term (i.e., days to a couple of weeks) responses to drought and flooding stress. A key hypothesis we propose this framework can test is that prior exposure to water stress alters future stress responses through the occurrence of a “memory,” which can be formed through both plant morphological changes and preconditioning of hormone levels. For instance, flooding‐induced aerenchyma formation may promote plant tolerance to subsequent drought due to a reduction in root metabolic cost (Klein et al., 2020), yet may simultaneously impede root water transport through the cortex (Yang et al., 2012). Aerenchyma formation can also reduce ABA biosynthesis because ABA biosynthesis can occur in parenchyma cells (Brunetti et al., 2019), which undergo apoptosis during aerenchyma formation. The ROL barrier formed under flooding can also improve plant tolerance to drought by reducing radial water loss to the dry soil (Song et al., 2022). Importantly, hormonal and physiological crosstalk is highly tissue and species‐specific. As an example, it is found that an ethylene pre‐treatment enhances ROS scavenging capacity and thereby tolerance to ROS‐mediated oxidative stress in roots (Peng et al., 2014; Liu et al., 2022). On the contrary, in aerenchyma‐forming species, ethylene reduces ROS scavenging to enhance ROS levels and induce aerenchyma formation in specific cell types (Steffens et al., 2011). It is exactly this complexity that makes predicting plant responses and their potential dependence on prior conditions and soil moisture conditions across the full spectrum from drought to flooding impossible without modeling the processes presented in our mechanistic framework.

A possible future extension of the mechanistic framework is to incorporate the life history of plants and the changes in plant architecture and physiology this results in, thus enabling the simulation at longer time scales, say months to years or even decades. Besides this extension on the temporal scale, we also envision straightforward extensions on the spatial scale. This proposed framework can serve to represent individuals within individual‐based models that scale up to the community and ecosystem level. Incorporating only horizontal water transport would already enable one to investigate competition for water and the impact of species composition and distribution on ecosystem water stress responses. Additionally, these models can be extended to incorporate, for example, interplant differences in their efficiency of nutrient and light acquisition and their competition for these resources.

A natural application for our model framework is its incorporation into land‐surface models. So far, the incorporation of plant hydraulics into land‐surface models has been used to investigate the feedback between climate and vegetation in terms of water cycle during non‐stressed conditions and drought, yet mechanistic plant responses to excessive soil water content are still lacking in these models (Li et al., 2021; Nguyen et al., 2020). With climate change expected to enhance surface evaporation and atmospheric vapor accumulation, the intensity and duration of droughts as well as the chances of flooding are both increased, underlining the demand for a framework capable of integrating both drought and flooding responses of plants (Trenberth, 2011). Therefore, through the incorporation of the model framework proposed here or simplifications thereof into larger‐scale land‐surface models, we aim to contribute to the improvement of our understanding of how plants help shape the climate.

## CONFLICT OF INTEREST STATEMENT

All co‐authors have no conflict of interest to declare.

## Data Availability

The authors confirm that the data supporting the findings of this study are available within the article.
